# Ancestry, race and ethnicity: the role and relevance of language in clinical genetics practice

**DOI:** 10.1136/jmg-2023-109370

**Published:** 2023-11-29

**Authors:** Melody Grace Redman, Rachel Helen Horton, Helena Carley, Anneke Lucassen

**Affiliations:** 1 Yorkshire Regional Genetics Service, Leeds Teaching Hospitals NHS Trust, Leeds, UK; 2 Centre for Personalised Medicine, Nuffield Department of Medicine, Wellcome Trust Centre for Human Genetics, Oxford, Oxfordshire, UK; 3 South East Thames Regional Genetics Service, Guy’s Hospital, London, UK

**Keywords:** genomics, human genetics, ancestry, ethnicity, race

## Abstract

**Background:**

The terms ancestry, race and ethnicity are used variably within the medical literature and within society and clinical care. Biological lineage can provide an important context for the interpretation of genomic data, but the language used, and practices around when to ascertain this, vary.

**Methods:**

Using a fictional case scenario we explore the relevance of questions around ancestry, race and ethnicity in clinical genetic practice.

**Results:**

In the UK, data on ‘ethnicity’ are routinely collected by those using genomic medicine, as well as within the wider UK National Health Service, although the reasons for this are not always clear to practitioners and patients. Sometimes it is requested as a proxy for biological lineage to aid variant interpretation, refine estimations of carrier frequency and guide decisions around the need for pharmacogenetic testing.

**Conclusion:**

There are many challenges around the use and utility of these terms. Currently, genomic databases are populated primarily with data from people of European descent, and this can lead to health disparities and poorer service for minoritised or underserved populations. Sensitivity and consideration are needed when communicating with patients around these areas. We explore the role and relevance of language around biological lineage in clinical genetics practice.

**WHAT IS ALREADY KNOWN ON THIS TOPIC**

- People are asked to answer questions about ethnicity in many walks of life.
- In the UK this might range from filling in census information, through job applications to medical encounters.
- The reason such questions are asked are not always clear and might be in attempts to measure both sociodemographic as well as ancestral diversity.

**WHAT THIS STUDY ADDS**

- We explored the relevance of questions about ethnicity in the context of genetic or genomic testing, and discuss how the reasons for the question may be unclear to both patient and clinicians and that the language used often compounds this.

**HOW THIS STUDY MIGHT AFFECT RESEARCH, PRACTICE OR POLICY**

- We highlight the skewing of current understanding of global genetic variation, such that genetic ancestry questions are important in interpretation of tests.
- However, the terms used do not map onto the answers being sought.
- We highlight the need for clarity and sensitivity, and explore the role and relevance of language around biological lineage in clinical genetics practice.

## Background

People are asked to answer questions about ethnicity in many walks of life. In the UK this might range from filling in census information, through job applications to medical encounters. The reason such questions are asked are not always clear and might be in attempts to measure both sociodemographic as well as ancestral diversity. We explored this question in the setting of genetic/genomic testing and discuss how the reasons for the question may be unclear to both patients and clinicians and that the language used often compounds this.

We start with a fictional clinical scenario to illustrate the issues in this paper: *A woman in her 30s, Ms AB, attends a Clinical Genetics consultation to discuss her diagnosis of polycystic kidney disease and is offered whole genome sequencing (WGS) to analyse a panel of relevant genes. As part of the consultation, she is asked to state her ethnicity. She asks the clinician why this is relevant. Her clinician is unsure how to respond*.

Differences in phenotypical appearances, particularly skin colour, have contributed to a damaging belief that there are substantial genetic differences between people with different geographical backgrounds. Only around 0.1% of human DNA varies between people, but this includes some 4.1–5 million variations in each individual.^1^ Most of this variation is shared among global populations but some genomic variants may be rare in particular populations and more common in others (perhaps reflecting ancestral geographical isolation). Knowing whether a variant is truly rare across the global population, or rare only in particular populations, may be helpful in deciding whether it is important in disease aetiology, and thus in understanding and diagnosing pathology.^2^ However, the language of ethnicity, and related terms, often conveys more than biological lineage and exploring the role and relevance of these terms is therefore important.

## Imprecision of language: terminology around ancestry, race and ethnicity

Before we can understand why the clinician asked about *Ms AB*’s ethnicity, we reflect on how terms ancestry, race and ethnicity are used variably throughout the medical and scientific literature and how these have changed during recent history. While the term ‘race’ appears frequently in American medical literature,^3^ in the UK’s National Health Service (NHS), the term ‘ethnicity’ is more commonly used to ask the same question. In our fictional case the term is used as a proxy to denote biological/genetic lineage rather than socioeconomic or cultural factors.

### Ancestry

In the Oxford English dictionary, ancestry is defined as: ‘the family or the group of people that you come from’.^4^ Data on ancestry may provide helpful insights into an individual’s genomic heritage, which we refer to as their ‘genomic ancestry’. Individuals may be able to recite their immediate ancestry—such as their parents and grandparents, however they may not be aware of their ancestry beyond recent generations, although they might be able to say that their ancestors came mainly from a particular land mass—for example, Chinese ancestry.

With increasing globalisation and movement of individuals across geographical boundaries, such information can become more challenging to obtain. Commercially available kits are sold as estimates of genomic ancestry, but can at best tell a customer where people with similar genetic variants currently live, rather than define geographical ancestry.^5^ These tests have been hugely popular. By 2019, over 26 million individuals were estimated to have accessed direct-to-consumer ancestry testing,^6^ although consistency of results between different companies is variable.^7^ Individuals taking up these tests are likely to interpret the concept of ancestry, at least to some degree, as a measure of their ancestors’ recent migration across the globe.

### Race

Race has been defined as ‘one of the main groups that humans can be divided into according to their physical differences, for example the colour of their skin…’ or as ‘a group of people who share the same language, history, culture, etc.’.^8^ Historically race was used in the wider scientific literature to delineate what were perceived to be different biological groups. However, race—like ethnicity—is better thought of as a complex social construct. This does not mean it is not important or that it should be ignored. However, there is extremely limited use for the word race in identifying genomic differences, as it is not a robust biological proxy. Additionally, in the UK, the use of the word race in a clinical context is tied to societal links with racism and colonialism.

### Ethnicity

Ethnicity has been defined as ‘the fact of belonging to… a group of people that share a cultural tradition’,^9^ or ‘a group of people who have a shared sense of identity because they have their own cultural background, traditions, history, language, etc.’.^10^

Ethnicity may provide a proxy of biological lineage in certain contexts but the term encompasses far more than biological lineage. However, in England’s genomic medicine service, ‘ethnic group’ is the category asked for on genomic test request forms, presumably as a proxy for biological lineage. Birney *et al* suggest that where ‘ethnicity’ is used, its context and the appropriate way to use it should be explicit, noting that some categories of ethnic groups such as ‘native American’ are sociopolitical terms which may not reflect ancestry.^11^ Importantly ethnicity cannot be judged by the clinician and is what the patient states it to be.

Any or all of these three terms—ancestry, race and ethnicity—can be of great personal significance and an important part of someone’s identity, but the often ambivalent use of these terms will not necessarily supply the information needed for variant interpretation in clinical genomic practice. Popejoy *et al*’s survey of American Clinical Genetics professionals found that the perceived definition and usefulness of the terms race, ethnicity and ancestry were variable, but at the same time this information was felt to be important for interpretation or communication around genetic testing.^12^ Interestingly, 27% of respondents to the survey felt ‘not at all’ confident in their ability to distinguish between the terms ethnicity, race and ancestry in general.^12^ Ancestry was felt to be the most ‘important’ term (out of race, ethnicity and ancestry) but at the same time no easier to obtain meaningful answers to in *Ms AB*’s situation.^12^ It is therefore not surprising that clinicians struggle with when and how to seek answers to information seen as essential on the laboratory request form. Recognition of significant limitations of the use of these terms in guiding clinical practice needs greater attention.

## Clinical relevance: why is ethnicity documented for genomic testing?

In *Ms AB*’s case, the main relevance of ethnicity to the genomics service is to inform variant interpretation. In a variety of clinical situations, ethnicity may also be relevant for genomic testing as part of carrier frequency estimation, pharmacogenetic testing, and Polygenic Risk Score application and interpretation.

### Informing variant interpretation

When requestingWGS through the NHS England genomics unit, clinicians are asked to input data on ethnicity onto the test request form.^13^ How these data are collected is at the requesting clinician’s discretion. As testing for ‘Cystic renal disease’ is performed using WGS (NHS England,^14^ p.352), *Ms AB*’s clinician will be asked to declare her ethnicity on the request form. It is important to note that ethnicity has no objective measure or score, and there are no universally agreed ethnic categories. Ethnicity entails much more than the phenotype observable by the clinician, and should not be assumed based on any factors such as appearance, name, skin colour or place of birth. Patients should be given the opportunity to self-report their ethnicity according to a category that they feel is the most appropriate.

The internationally accepted American College of Medical Genetics and Genomics guidelines for variant interpretation involve establishing the presence or absence of the variant in a ‘race-matched’ population.^3^ This proxy may affect the level of evidence applied regarding the pathogenicity of a variant (with common variants being dismissed as a likely cause of rare disease on the premise that if they did cause disease, the disease would be more common).

In analysis of genomic variation, those variants found in an individual would be screened against appropriate reference data sets. These data sets include participants categorised in terms of biological ancestry. For example, the widely used gnomAD^15^ database uses ‘super-population’ ancestry including categories such as ‘African/African American’ or ‘East Asian’, with some subcontinental ancestry information provided such as ‘Japanese’.^16^ However, these categories may be poorly defined, used differently by different groups, and are not necessarily representative of biological diversity. Individuals may have more genetic similarity with a member of a different super-population than individuals from within their own.^17^ For example, the greatest level of genetic diversity in the world is found within Africa.^18^ This is further complicated by recording an individual’s ethnicity as ‘African’ as this does not allow a granular understanding of genomic ancestry.

### Refining estimation of carrier frequency

For some conditions, a person’s ethnicity is considered to assist with the calculation of prior probability of disease likelihood using Bayes theorem.^19^ In this context, the frequency of disease alleles within the specified ‘ethnic’ group is needed to improve the accuracy of the estimation. Isolated island populations or communities may have increased frequencies of specific variants, leading to increased prevalence of certain genetic conditions. Such a ‘founder effect’ is described as a genetic variant frequently observed in a group due to geographical or cultural isolation. For example, the carrier frequency of Gaucher disease in individuals with Ashkenazi Jewish heritage is reported to be approximately 1 in 18 due to the founder effect,^20^ compared with 1 in 125 to 1 in 143 in a non-Jewish population.^21^ Among the Croatian Islands, familial ovarian cancer is frequently observed on Lastovo island while Mal de Meleda (a rare skin condition) is more frequently observed in Mljet island.^22^ This variation in frequencies means that the reproductive risk advice needs to be tailored appropriately.

We know that carrier frequencies can vary widely across ethnicities.^23^ This may affect patients’ ability to access carrier testing in England—for example, guidance in England currently advises that carrier testing for an autosomal recessive condition should be offered to partners of known carriers if the carrier frequency is higher than 1 in 70 for the relevant population (14, p. 399). However, carrier frequencies for particular populations may not be known since population studies are needed to predict carrier frequencies accurately. This means that people from ethnic groups who are under-represented in existing databases may not be able to access carrier testing because there are insufficient data available.

### Guiding decisions around the need for pharmacogenetics testing

Pharmacogenomics explores the relationship between genomic variation and drug effects. For example, people with the HLA-B*5801 allele are known to be at increased risk of developing allopurinol hypersensitivity syndrome, and potentially life-threatening Stevens-Johnson syndrome, when they take a widely used urate-lowering drug called allopurinol. Current UK guidance recommends that screening for this allele should be considered in individuals from ethnic groups where the prevalence of the HLA-B*5801 allele is known to be high.^24^ This includes individuals of Korean, Han Chinese and Thai descent, where this allele is found in 6%–12% of people.^24^ US guidance also advises testing for this allele within these groups, and also for African-American patients.^25^ In this context, ethnicity is used to stratify risk and therefore prioritise testing of particular groups. One possible change to this process could be to test all individuals for the HLA-B*5801 allele before starting allopurinol, but there would be significant resource and cost implications for such a decision.

### Interpreting Polygenic (Risk) Scores

Polygenic Risk Scores seek to measure the combined effect of many different genetic variants on a person’s risk of developing relatively common conditions such as diabetes, heart disease or cancer. Creation of such scores relies on large Genome Wide Association Studies (GWAS), which aim to identify common genetic variants which influence predisposition to disease. However, scores work best for people whose genetic variations are well represented in data sets. For people whose genetic data is not well represented, the scores stand to perform poorly. As of February 2023, 95.2% of the participants who had contributed to GWAS were ’European’.^26^ Without adequate study of different population-based allele frequencies, it is difficult to know the association of particular SNPs with the disease.^27^ Martin *et al* illustrate poor performance of multiple PRS across non-European cohorts, which they surmise is because the study populations were based on European GWAS cohorts. For example, when comparing 17 quantitative anthropometric and blood panel traits, the authors found the prediction accuracy was 4.9-fold lower in African populations and 2.5-fold lower in East Asian populations.^28^ Similarly, Duncan *et al* found that PRS performance was worst among those with African ancestry, with a median effect size only 42% compared with matched samples from individuals of European ancestry.^29^ The performance of PRS tools varies dependent on a person’s ancestry, and if an individual’s ancestry was not adequately represented in genomic data sets, this can potentially lead to increasing health disparities.

## The use of ethnicity data within the wider UK NHS

While *Ms AB*’s ethnicity is sought in the Clinical Genetics setting for its apparent clinical utility, these data may also be recorded elsewhere in the NHS for different purposes. For example, NHS Digital collects information on ethnicity from Hospital Episode Statistics and general practice databases using ethnic categories that have been set by the UK Office for National Statistics in the 2011 census. These data are used for a range of purposes including data sets on hospital episodes, workforce and commissioning.^30^ For example, these data have been used to study the susceptibility of individuals from ‘Black, Asian, Mixed-race and Ethnic minorities’ to severe COVID-19 disease.^31^

It is too simplistic to link health outcomes to ethnicity data without considering confounding socioeconomic factors, although this is for a different reason than understanding biological lineage for the purposes of genomic information interpretation.^32^

Polubriaginof *et al* argued that until we achieve health equity, it may still be necessary to collect data on the social determinants of health—including race and ethnicity.^33^ These data may be helpful to identify disadvantaged minority groups although each person fitting into a particular (poorly defined) category will not have the same level of advantage/disadvantage, since many other factors—such as education and occupation—affect health equity.^34^

## Issues around the use of ancestry, race and ethnicity in genomics

Several questions remain around the appropriate use of the term ‘ethnicity’ in genomics. If ethnicity data are used to inform genomic interpretation, and there is a lack of understanding of the terms ancestry, race and ethnicity, are we perpetuating the idea of these terms as a biological construct? There is reason to be cautious: as recently as 2018, the American Society of Human Genetics issued a statement rejecting genetic variation as a mechanism to shore up ideas of racial supremacy in response to the misuse of genomic research by white supremacists.^35^ Instead of asking questions about ethnicity when attempting to infer biological lineage, data sets with ancestry inferred markers to extrapolate information on genetic ancestry may be a helpful alternative, but again the over-representation of those from European ancestry leads to limitations.^36^ A further consideration is that ethnicity can include the sharing of environmental factors which might affect gene expression.

There are disparities in the utility of genomic testing in groups of different recorded ancestry; despite the limitations of the term, these disparities do indicate some need to seek equity for different groups. The *Deciphering Developmental Disorders* study found a lower diagnostic rate (OR=0.51; 95% CI 0.31 to 0.78) in those with African ancestry than those with other ancestries.^37^ The main contributing factor was the high proportion of ‘singleton’ exomes submitted in participants of African ancestry. Singleton exomes are more challenging to interpret than ‘trio’ exomes (in which rare genetic variants in an offspring’s sample may be filtered against samples from healthy parents to identify likely benign variations).^37^ Additionally, fewer variants were able to be filtered out for non-European cases; likely due to difficulties in estimating allele frequencies due to a lack of appropriate controls.^37^ It is also widely acknowledged that phenotypical features—especially facial dysmorphism—are less well described in those of African ancestry.^37 38^

Major work is going on to address the lack of diversity in genomic data. It is widely accepted that health disparities and unmet health needs are accentuated by a lack of diversity in genomic research.^39^ Most genomic studies (86%) have been conducted using data from individuals of European ancestry (as of June 2021).^39^ The Human Pangenome Project seeks to produce a reference genome which better represents global human genomic diversity.^40^ Genomics England have introduced a ‘Diverse Data’ initiative to seek to improve research, prognosis, diagnosis, treatment and trust across diverse populations.^41^ Hardcastle *et al* have published a detailed literature review and synthesis on the ethical, legal and social issues in diversifying genomic data.^32^

Fatumo *et al* demonstrate many examples of successful genomic research conducted in under-represented groups.^39^ Key features for success include sufficient strategic funding and support for researchers at institutions in low-income and middle-income countries.^39^ Most countries recognised as low-income and middle-income are non-European and their populations are poorly represented in genomic datasets.^42^ Further investment and targeted support for genomic research is needed to reduce the impact of data disparities that currently exist. Increased diversity in genomics needs to extend beyond data capturing and become embedded in all aspects of practice. Careful consideration should be given to language used to describe groups of people to ensure that individuals are not, or do not feel, excluded from the benefits of genomic medicine.

### Impact on patients

Ethnicity is the most widely used term to denote biological lineage in the UK. However, many factors—including biological, social, cultural, religious and genetic factors—may influence someone’s ethnicity. Patients may find that the categories of ethnicity they are asked to choose from do not allow sufficient description of their situation. An individual’s stated ethnicity may vary depending on the context of the question and the purpose of the information. Geographical origin—such as the continent on which an individual or their parents were born—may not reflect the genetic factors from previous generations. Genealogical mapping tools have helped observe the complex migration of humans and how individuals across the globe are related to each other,^43^ demonstrating that clear delineation of ancestral lineage is not possible.

A 2019 Canadian study found that patients understood different things when asked about race and ethnicity by their family doctor although they did not mind the question in itself.^44^ For example, some felt the question related to their place of birth and some felt it related to their parents’ ancestry. The response options that were available influenced how individuals self-identified, and patients found it particularly difficult if they felt they belonged to more than one group.^44^ However, a 2005 study conducted in the USA found that many patients feel uncomfortable about providing information on race/ethnicity, but most patients do think it is appropriate for healthcare professionals to collect this information.^45^ There were concerns that this information may be used for patient discrimination.^45^ It may therefore be unclear to patients such as *Ms AB* why their ethnicity is medically relevant. Patients may not be aware of the lack of diversity in genomic data sets, and individual clinicians may differ as to if, and when they mention this. It might be considered an important aspect of genetic counselling, for example, when discussing the likelihood of obtaining a variant of uncertain significance. It is important to recognise that ethnicity might intersect with other identities and characteristics, compounding feelings of marginalisation for some patients.

The presence of a question around ethnicity may raise suspicion due to historic examples of medical maltreatment aligned with patient ethnicity (such as the Tuskegee Study of Untreated Syphilis), which have contributed to a level of mistrust towards healthcare professionals and researchers. This wider sense of mistrust may contribute to a reluctance from patients to share data on ethnicity or to contribute to genomic research.^46^

We must be mindful of other concerns that individuals might have about ethnicity data being collated. Genetic essentialism (the belief that an individual’s behaviours and characteristics are explained by their genes) may be used in some settings to inappropriately justify inequalities within different ethnic groups.^47^ Individuals may perceive a genetic condition as being their ‘fault’ because of their ethnicity. There may be fear of stigmatisation if the purpose of the question is not made clear, or due to the language used by the clinician, or the patient’s understanding of terminology. If patients do not understand the relevance of being asked about their ethnicity, it may affect the uptake of genetic testing from underserved communities, which is already seen—for example in women from minority ethnic groups who are less likely to undergo *BRCA* testing.^48^

## Conclusion

Information on biological lineage is of relevance to clinical geneticists to inform variant interpretations, refine estimations of carrier frequency, guide decisions around the need for pharmacogenetic testing, and the utility of Polygenic Risk Scores. However, the use of language to ascertain this information is imprecise and problematic, and biological lineage is one of many factors that may influence someone’s identity. While most human DNA is shared between all people, the small percentage that varies between individuals will sometimes be important in affecting an individual’s development and health. Genomic datasets historically focus heavily on individuals with European ancestryand this can exacerbate health disparities and unmet health needs for under-represented populations.

Returning to our fictitious case, *Ms AB* is asked about her ethnicity as part of the WGS request form. As mentioned, one reason is to facilitate risk calculation and interpretation of genomic data—this may have personal implications for the patient; but second to assess equity of access to genomic services—this is more relevant for wider society. For risk calculation and interpretation of genomic data, the role of recording ethnicity is as a proxy for biological lineage to select the most suitable population as a comparator for data interpretation, acknowledging the limitations of current genomic data sets.

Further work is needed to understand the experiences of patients and healthcare professionals around the categorisation of human variation and diversity, and the language used to describe this in UK clinical genetics practice. It may be that as global populations mobilise and as more diverse populations are included in genomic reference data sets, questions around ethnicity may lose their potential clinical significance, although efforts to diversify data sets to date have fallen short.^49^ In the medium term, ancestry inferred markers through SNP tools may have a role, although work would be needed to explore if this would be of benefit within UK practice.

The role of recording ethnicity in wider UK healthcare for sociodemographic reasons such as assessing equity of access to genomics is relevant from a social perspective, however, must be interpreted in the context of the many confounding factors which may influence an individual’s access to services. In this setting, it is important that an individual can identify into the group that they feel is most relevant for them. Some form of categorisation for this purpose may be necessary, but there are further questions about the appropriate range of categories that should be offered, and how, when and where this question should be asked.

## Data Availability

No data are available.
